# Diffusion of excellence: evaluating a system to identify, replicate, and spread promising innovative practices across the Veterans health administration

**DOI:** 10.3389/frhs.2023.1223277

**Published:** 2024-02-13

**Authors:** George L. Jackson, Gemmae M. Fix, Brandolyn S. White, Sarah L. Cutrona, Caitlin M. Reardon, Laura J. Damschroder, Madison Burns, Kathryn DeLaughter, Marilla A. Opra Widerquist, Maria Arasim, Jennifer Lindquist, Allen L. Gifford, Heather A. King, Jenesse Kaitz, Guneet K. Jasuja, Timothy P. Hogan, Jaifred Christian F. Lopez, Blake Henderson, Blaine A. Fitzgerald, Amber Goetschius, Danielle Hagan, Carl McCoy, Alex Seelig, Andrea Nevedal

**Affiliations:** ^1^ Center of Innovation to Accelerate Discovery and Practice Transformation (ADAPT), Durham Veterans Affairs (VA) Health Care System, Durham, NC, United States; ^2^ Advancing Implementation and Improvement Science Program, Peter O’Donnell Jr. School of Public Health, University of Texas Southwestern Medical Center, Dallas, TX, United States; ^3^ Center for Healthcare Organization & Implementation Research, Bedford & Boston VA Medical Centers, Bedford and Boston, MA, United States; ^4^ Section of General Internal Medicine, Boston University Chobanian and Avedisian School of Medicine, Boston, MA, United States; ^5^ Department of Health Law, Policy & Management, Boston University, Boston, MA, United States; ^6^ Division of Health Informatics and Implementation Science, Department of Population and Quantitative Health Sciences, University of Massachusetts Chan Medical School, Worcester, MA, United States; ^7^ Center for Clinical Management Research, VA Ann Arbor Healthcare System, Ann Arbor, MI, United States; ^8^ Department of Population Health Sciences, Duke University, Durham, NC, United States; ^9^ Division of General Internal Medicine, Duke University, Durham, NC, United States; ^10^ VHA Innovation Ecosystem, Office of Healthcare Innovation and Learning, United States Veterans Health Administration, Washington, DC, United States; ^11^ Agile Six Applications, Inc., San Diego, CA, United States

**Keywords:** implementation science, innovation, program evaluation, RE-AIM, Veterans

## Abstract

**Introduction:**

The Veterans Health Administration (VHA) Diffusion of Excellence (DoE) program provides a system to identify, replicate, and spread promising practices across the largest integrated healthcare system in the United States. DoE identifies innovations that have been successfully implemented in the VHA through a Shark Tank style competition. VHA facility and regional directors bid resources needed to replicate promising practices. Winning facilities/regions receive external facilitation to aid in replication/implementation over the course of a year. DoE staff then support diffusion of successful practices across the nationwide VHA.

**Methods:**

Organized around the Reach, Effectiveness, Adoption, Implementation, and Maintenance (RE-AIM) Framework, we summarize results of an ongoing long-term mixed-methods implementation evaluation of DoE. Data sources include: Shark Tank application and bid details, tracking practice adoptions through a Diffusion Marketplace, characteristics of VHA facilities, focus groups with Shark Tank bidders, structured observations of DoE events, surveys of DoE program participants, and semi-structured interviews of national VHA program office leaders, VHA healthcare system/facility executives, practice developers, implementation teams and facilitators.

**Results:**

In the first eight Shark Tanks (2016–2022), 3,280 Shark Tank applications were submitted; 88 were designated DoE Promising Practices (i.e., practices receive facilitated replication). DoE has effectively spread practices across the VHA, with 1,440 documented instances of adoption/replication of practices across the VHA. This includes 180 adoptions/replications in facilities located in rural areas. Leadership decisions to adopt innovations are often based on big picture considerations such as constituency support and linkage to organizational goals. DoE Promising Practices that have the greatest national spread have been successfully replicated at new sites during the facilitated replication process, have close partnerships with VHA national program offices, and tend to be less expensive to implement. Two indicators of sustainment indicate that 56 of the 88 Promising Practices are still being diffused across the VHA; 56% of facilities originally replicating the practices have sustained them, even up to 6 years after the first Shark Tank.

**Conclusion:**

DoE has developed a sustainable process for the identification, replication, and spread of promising practices as part of a learning health system committed to providing equitable access to high quality care.

## Introduction

All healthcare systems ultimately seek to provide equitable access to the highest quality of care possible within the confines of available resources (1–3). Laid bare by the realities of the COVID-19 pandemic, these goals can only be accomplished though well-functioning organizations that also address the needs of staff such as addressing staff well-being and team function (4–8). Additionally, there has been increasing awareness of the importance of taking a population health approach that considers how the services provided by healthcare systems impact the health of individual patients, caregivers, communities, and the population more broadly (9–13).

To further their mission, many healthcare organizations have sought to become learning health systems. In learning health systems, teams have both research/evaluation and quality improvement expertise and seek to utilize a combination of data, improvement, and implementation science and practice to identify, implement, and evaluate opportunities to address health system challenges (14–21). In addition to addressing the day-to-day challenges of healthcare operations, these learning health systems incorporate the insights of frontline staff in combination with scientific methods to develop and test innovative solutions to address the challenges facing healthcare systems.

Numerous large healthcare systems have established innovation programs, centers, or events (e.g., Shark Tanks) to develop healthcare innovations. These innovations range from enhancement of clinical service delivery to efficient management of administrative needs to the production of new healthcare devices or computer applications to address specific patient challenges (22–28). These innovation centers and programs are not simply research centers by another name. Research centers seek to produce new, generalizable knowledge that has broad applicability regardless of the specific healthcare system. The innovation centers/programs often collaborate with researchers and may produce innovations with wide applicability. However, a focus of innovation centers/programs is on enhancing the care and services offered at a specific healthcare system. While the precise objectives and size of innovation efforts vary, key steps include the following: (1) providing opportunities to develop new innovations within the organization (often by frontline staff); (2) identifying promising practices that have worked in similar settings that may be adopted elsewhere; (3) piloting the use of innovations; (4) developing strategies to support implementation of successful innovations; (5) diffusing successful innovations across what are often very large healthcare systems; (6) evaluating the degree of innovation adoption and impact across the healthcare system; and (7) supporting the long term sustainment of innovations (14, 29–31).

The VHA Diffusion of Excellence (DoE) program represents a process to identify, replicate, and spread promising practices across the largest fully-integrated healthcare system in the United States, the Veterans Health Administration (VHA) (30, 32–42). The present paper summarizes findings from an embedded, mixed-methods, theory-based evaluation of DoE with results summarized according to the Glasgow RE-AIM (Reach, Effectiveness—Adoption, Implementation, Maintenance) implementation evaluation framework (43–46). Specifically, this paper presents the evaluation of the methods utilized by DoE to identify, replicate, and spread a wide range of innovative practices, as opposed to focusing on the clinical or administrative effectiveness of a specific innovation. Findings are organized based on DoE program: (1) reach (degree of participation in DoE); (2) effectiveness (spreading of promising practices across the VHA and summary of patients served by individual DoE practices); (3) adoption (considerations relating to adopting practices and participating in DoE); (4) implementation (factors influencing implementation success at new facilities); and (5) maintenance (sustainment of DoE supported innovations).

## Materials and methods

### Setting: the Veterans health administration (VHA)

The VHA is the largest fully integrated healthcare delivery system in the United States. It offers a full range of primary, mental health, and specialty care and takes a population-based approach to addressing the health needs of Veterans. Services also address social determinants of health (e.g., identifying and addressing homelessness). Additionally, the VHA has an extensive medical and health research program, the largest health professions training program in the United States, and plays a significant role in emergency preparedness and disaster relief (47).

In 2022, the VHA provided care to 6.75 million patients through the work of over 341,000 employees at more than 1,200 sites of care. Approximately 9% of VHA patients are women and almost one-third reside in rural areas. Veterans who utilize the VHA tend to be of lower income and have greater health challenges than other Veterans and the general population (48). In sum, the VHA has a major impact on the lives of millions of people each year with a broad range of health related roles and operations than can benefit from innovation.

The VHA has 18 regions called Veterans Integrated Service Networks (VISNs). Nationwide, the VISNs contain a total of 141 parent healthcare systems/facilities. The term “parent” refers to the fact that these parent healthcare systems/facilities contain individual treatment locations, such as hospitals and community-based outpatient clinics. The parent healthcare system/facility has a single Director and associated leadership team that is responsible for services provided at each of the associated individual treatment locations under their purview. In total there are 1,254 individual treatment locations across the VHA. Treatment locations are in all states and territories of the United States, along with the Philippines.

### Program: VHA diffusion of excellence (DoE)

The program was started in 2015 with the goal of identifying and spreading promising practices that can enhance the quality of services provided to Veterans across the entire VHA. DoE is currently part of the VHA Innovation Ecosystem, which sits within the VHA Office of Healthcare Innovation and Learning (OHIL). In 2016, DoE launched a VHA Shark Tank competition with the goal of learning about promising practices that have had a positive impact at local sites of care in the VHA and then supporting replication of the practices at new locations.

### Shark tank competition: selection of promising practices

Frontline VHA staff (e.g., clinicians caring for Veterans; administrative staff) apply to pitch their successful promising practices addressing high-priority topics to VHA parent healthcare system/facility and regional directors (i.e., “Sharks”). Priorities reflect the overall priorities of the VHA at the time of the specific Shark Tank. For example, current (as of May 2023) priorities of the VHA Under Secretary for Health include: (1) hiring staff faster and more competitively (e.g., speeding up the onboarding process); (2) connecting Veterans to the soonest, best care (e.g., access, care coordination, referral coordination); (3) addressing the needs of Veterans with military environmental exposures; (4) accelerating VHA's journey to a high reliability organization (e.g., enhancing quality of care); (5) supporting Veterans' whole health, including needs of caregivers and survivors; and (6) prioritizing suicide prevention. While the exact wording may change over time, VHA priorities tend to fall into these broad categories.

Eligible promising practices must have demonstrated a measurable positive impact in at least one VHA parent healthcare system/facility, with preference given to practices that have been successfully utilized in multiple locations. However, these innovations have a varying degree of traditional scientific evidence, ranging from an indication of success in one location all the way to controlled observational studies and randomized clinical trials (e.g., 49–51). As a result, promising practices can be termed “evidence-informed” (52). Shark Tank applications are judged based on the degree to which the proposed innovative practice: (1) aligns with VHA priorities/strategic objectives; (2) solves a specific problem that is of importance to Veterans, VHA constituents, or the VHA as a whole; (3) includes data that indicates a positive impact on the problem and satisfaction of Veterans or targeted individuals (e.g., VHA staff or Veteran caregivers); (4) describes resource requirements (e.g., equipment, staff time) and (5) provides evidence that it can be replicated in a new parent healthcare system/facility in less than 12 months. None of these criteria have greater *a priori* weight than others.

Both subject-matter experts and frontline staff review all applications. Approximately 100 semifinalists are selected per Shark Tank cohort. Starting with the fourth Shark Tank, semifinalists are also reviewed by representatives from the VHA Quality Enhancement Research Initiative (QUERI) (17, 53) (not part of the present evaluation described in this paper) who rate practices based on evidence, feasibility, potential impact, and clinical soundness. Throughout the selection process (i.e., application to semifinalist to Shark Tank finalist) information from reviewers is utilized by DoE and Innovation Ecosystem leadership to make final decisions about the application. This is similar to other peer review processes such as editors making final decisions about the publication of papers based on reviewer comments or grant agency staff making final decisions about what grants are funded based on peer-review information.

Approximately 15–20 finalists are selected to pitch their ideas during the annual Shark Tank competition. The format of the Shark Tank has changed over the years. Initially, it was a live, virtual event in which participants gave short pitches that addressed the problem, innovative solution, impact, and needed resources. Sharks placed bids in real time, committing resources to support projects (e.g., personnel time, travel support). Winning bids received facilitation to guide implementation of the designated practice in their parent healthcare system/facility or region. In 2019, a hybrid format was used; Sharks could bid resources live or virtually. With the COVID-19 pandemic, a virtual system was again used. Starting with the 7th Shark tank in 2021, the bidding process changed substantially. Instead of Sharks bidding in real time, descriptions of each proposed practice were made available in advance. Sharks were asked to both indicate the need for the practice within their parent healthcare system/facility or region and indicate what resources will be made available to support the replication of the innovative practice.

Shark Tank bids are reviewed by leadership of the Office of Healthcare Innovation and Learning, Innovation Ecosystem, and Diffusion of Excellence. Winning bids and related promising practices are selected based on content of the bid, fit with the parent healthcare system/facility or region, and potential value of the practice. In the most recent two Shark Tanks, it was decided that one additional winner could be selected by popular vote of people observing live Shark Tank pitches. For each selected practice, typically between one to three parent healthcare system/facilities win the opportunity to receive external facilitation to implement the winning practice (known as DoE Promising Practices) within approximately six to 12 months. Table 1 contains examples of selected practices.

**Table 1:** Diffusion of excellence national diffusion practices that receive with direct assistance of diffusion of excellence staff diffusion specialists.

Practice name	Brief description
Selected as national diffusion practice in 2018—from winners of the 2nd VHA Shark Tank	
HAPPEN (Hospital-Acquired Pneumonia Prevention by Engaging Nurses)	Originating facility and DoE fellow: Salem VA Medical Center (Salem, Virginia)—Shannon Munro
	Number of times adapted by VHA facilities/sites of care: 155 Community Living Centers (CLCs)/skilled-nursing facilities (100% of CLCs)
	Number of Veterans Served as of September 30, 2022: 277,692
	Summary from the VHA diffusion marketplace: HAPPEN, or Hospital Acquired Pneumonia Prevention by Engaging Nurses supports VHA priorities by reducing the risk of non-ventilator associated hospital acquired pneumonia (NV-HAP), improving the health and quality of life of our Veterans; modernizing systems and processes with a focus on preventive care; improving access and timeliness of service by reducing patient length of stays; and reducing health care cost.
VHA rapid naloxone initiative (Opioid overdose reversal through availability of intranasal (IN) naloxone to patients, VHA police, and within automated external defibrillator (AED) cabinets)	Originating facility and DoE fellow: VA Boston Health Care System (Boston, Massachusetts)—Pamela Bellino-Rivera
	Number of times adapted by VHA facilities/sites of care: 116 Health Care Systems (HCSs) [VA Police] (82% of HCSs); 56 HCSs (AED Cabinets) (40% of HCSs)
	Number of Veterans served as of September 30, 2022: 120 overdose reversals documented
	Police Officers equipped with intranasal naloxone as of September 30, 2022: 2,785
	AED Cabinets equipped as of September 30, 2022: 693
	Summary from the VHA diffusion marketplace: In September 2018, the Veteran's Health Administration (VHA) launched a Rapid Naloxone Initiative that aims to reduce opioid overdose deaths by increasing the rapid availability of Naloxone. This is done via three practice elements: (1) Opioid Overdose Education and Naloxone Distribution (OEND) to VHA patients who are at-risk for opioid overdose, (2) VHA Police carry Naloxone, and (3) Automated External Defibrillator (AED) Cabinets contain Naloxone.
TeleWound (Increasing wound care access for rural Veterans through telehealth)	Originating Facility and DoE Fellow: Mountain Home VA Medical Center (Mountain Home, Tennessee)—Mona Baharestani
	Number of times adapted by VHA facilities/sites of care: 38
	Number of Veterans served as of September 30, 2022: 2,333
	Summary from the VHA diffusion marketplace: The National TeleWound Care Practice delivers a national standard for sites to stand up new TeleWound programs. TeleWound Care brings specialty wound care to Veterans irrespective of where they are. Through TeleWound Care, early intervention is provided, to avoid complications such as increased infection and amputation.
Selected as national diffusion practice in 2018—from winners of the 3rd VHA Shark Tank	
FLOW3 (System provides accurate and reliable real-time data for process control and reporting of prosthetic consults)	Originating facility and DoE fellow: VA Puget Sound Health Care System (Seattle, Washington)—Jeffrey Heckman
	Number of times adapted by VHA facilities/sites of care: 138 Prosthetic Clinics (100%)
	Number of Veterans served as of September 30, 2022: 12,294
	Summary from the VHA diffusion marketplace: FLOW3 is a system built by a team of VA staff including physicians, prosthetists, prosthetics purchasing, contracting, and administrative staff that will transform the process for prosthetic limb care. It was designed to work alongside the VA Computerized Patient Record System (CPRS) using custom software, including the FLOW Consult Comment© application and a web-based application that will transition with VA from the CPRS to Cerner electronic health record.
VIONE—medication optimization and polypharmacy reduction initiative (A de-prescribing approach to medication management)	Originating facility and DoE fellow: Central Arkansas Veterans Health Care System's Eugene J. Towbin Healthcare Center (Little Rock, Arkansas—Sara Swathy Battar, Timothy Cmelik, & Kimberly Dickerson
	Number of times adapted by VHA facilities/sites of care: 123 successful, 7 in-progress
	Number of Veterans served as of September 30, 2022: 191,889
	Summary from the VHA diffusion marketplace: VIONE is a clinician and patient-friendly methodology—VIONE (Vital, Important, Optional, Not needed, Every medication has an indication) used by physicians, nurse practitioners, physician assistants, nurses, and pharmacists to address polypharmacy risk. VIONE is an option for review of medications consistent with Age Friendly 4 Ms. It has global relevance and offers a viable solution to polypharmacy while improving patient safety and quality of life. VIONE indirectly facilitates a greener environment and decreases preventable adverse polypharmacy related outcomes across the continuum of clinical care. It exemplifies concepts of High Reliability Organizations.
Selected as national diffusion practice in 2019—from winners of the 3rd VHA Shark Tank	
4-Sight (system to increase efficiency of eyeglass ordering)	Originating facility and DoE fellow: Tibor Rubin VA Medical Center (Long Beach, California)—David Hook, Brian Kaiser, Spencer Mion, Timothy Strebel, Pamela Westbrooks
	Number of times adapted by VHA facilities/sites of care: 65
	Number of Veterans served as of September 30, 2022: 337,186
	Summary from the VHA diffusion marketplace: Eyeglasses make up 30% of prosthetic purchases at VA. The time between an initial prescription and final delivery can greatly affect a Veteran. 4-Sight was developed to increase efficiency in eyeglass ordering. The program reduces the manual processes completed by prosthetics staff and encourages automated eyeglass consultations. 4-Sight leverages technology to automate actions in the VA's Veterans Health Information System Technology Architecture (VistA) computer system and ensures that vendors receive the patient prescription information exactly as it was entered by clinicians. This increases the likelihood that Veterans will receive the right eyeglasses the first time.
STRIDE—getting hospitalized veterans back on their feet (early walking for hospitalized Veterans)	Originating facility and DoE fellow: Durham VA Health Care System (Durham, North Carolina)—Susan Nicole (Nicki) Hastings
	Number of times adapted by VHA facilities/sites of care: 41 successful; 17 in-progress
	Number of Veterans served as of September 30, 2022: 2,876
	Summary from the VHA diffusion marketplace: STRIDE is a supervised walking program for older Veterans admitted to the hospital with medical illness. STRIDE features an early assessment, supervised ambulation, and patient education about the importance of daily walking, all designed to ensure patient safety during program participation. Developed with input from multiple disciplines, STRIDE fills an urgent need for promotion of early, safe mobility in hospitalized individuals to prevent negative consequences of inpatient bedrest and immobility.
Selected as national diffusion practice in 2020—from winners of the 3rd VHA Shark Tank	
Veterans Mental Evaluation Team [VMET] (crisis de-escalation, mitigate incarceration, expedite access to care)	Originating facility and DoE fellow: VA Long beach healthcare system (Long Beach, California)—Tyrone Anderson & Shannon Teague
	Number of times adapted by VHA facilities/sites of care: 6 successful; 2 in-progress
	Number of Veterans served as of September 30, 2022: 1,051
	Summary from the VHA Diffusion Marketplace: The VMET program assists in responding to calls involving local law enforcement interactions with veterans in crisis. In addition, the VMET team conducts outreach efforts to contact “at risk” veterans who have stopped showing up for their psychiatric care at the VA Hospital. This proactive outreach approach, which includes a VA clinician and VA police officer, is a first of its kind VA nationwide program to help reduce the number of veteran suicides and increase participation in mental health treatment. All veterans have access to follow up care and wrap around services.
Surgical pause (enhancing risk assessment and care for potentially frail patients undergoing surgery)	Originating Facility and Lead Developer: Omaha VA Medical Center (Omaha, NE)—Daniel Hall
	Number of times adapted by VHA facilities/sites of care: 44 successful; 33 in-progress
	Number of Veterans served as of September 30, 2022: 49,670
	Summary from the VHA diffusion Marketplace: Data demonstrate there is no such thing as low risk surgery in high risk, frail patients; in fact, 1 in 3 frail Veterans will die within 6 months of even “small surgery”. Historically, there was no reliable and quick way to identify the highest risk patients at the point of care before committing to a surgical plan. Recognized as a Promising Practice by the VHA Innovation Ecosystem's Diffusion of Excellence, the Surgical Pause utilizes the simple yet sophisticated Risk Analysis Index (RAI) to screen Veterans for frailty in 30 s, effectively flagging high risk Veterans so that the surgical team can ensure that the proposed treatment plans both mitigate known risks and align with the Veterans’ overarching life goals. Transparent communication about the risks of protracted recovery or loss of independence after surgery empowers patients to consider non-operative management strategies. And for those who nonetheless elect surgical intervention, preoperative exercise training for as little as 3–6 weeks before surgery may improve outcomes by increasing physiologic reserve. By bringing additional resources to such frail patients, the Surgical Pause improves outcomes and adds value.
Selected as national diffusion practice in 2020—from practices initially supported by the VHA innovators network	
PRIDE in all who served (Peer health education group for LGBTQ + Veterans)	Originating facility and lead developer: Hampton VA Medical Center (Hampton, Virginia)—Tiffany Lange
	Number of times adapted by VHA facilities/sites of care: 50 successful, 18 in-progress
	Number of Veterans served as of September 30, 2022: 236
	Summary from the VHA diffusion marketplace: The LGBTQ + Health Education Group was designed and refined using human centered design principles at the Hampton VA Medical Center in 2016. Each one-hour session of the ten-week, manualized, closed group provides an opportunity to connect with other LGBTQ + Veterans to build a peer support system while improving LGBTQ + patient health literacy. Connection to social support is a protective factor against suicide, stigma-related stress and other health outcomes for LGBTQ + individuals. The group facilitation manual provides a comprehensive approach to implementation of the service, staff training, and guidance on how to create a welcoming environment.
Selected as national diffusion practice in 2021—from winners of the 6th VHA Shark Tank	
Remote temperature monitoring (Reducing hospital admissions and amputation prevention among patients with diabetes)	Originating facility and DoE fellow: Cincinnati VA Medical Center (Cincinnati, Ohio)—Kyle Nordrum
	Number of times adapted by VHA facilities/sites of care: 95
	Number of Veterans served as of September 30, 2022: −3,600
	Summary from the VHA diffusion marketplace: One in four Veterans has diabetes. In 2021, VA treated 75,000 Diabetic Foot Ulcers (DFU), which accounted for 80% of non-traumatic amputations, resulting in a cost of $3.2 billion. VHAIE's “National Initiative to End Diabetic Limb loss”, has designed and tested a new care model in DFU prevention. Since COVID, we have shifted from traditional in-clinic treatment to virtual-based prevention.
PACT PT (physical therapy imbedded within primary care patient aligned care teams)	Originating facility and DoE fellow: Des Moines VA Medical Center (Des Moines, Iowa)—Chris Rowedder
	Number of times adapted by VHA facilities/sites of care: 59 successful, 48 sites near full implementation, 60 sites expected to be added in the summer of 2023
	Number of Veterans served as of September 30, 2022: 100,000+
	Summary from the VHA diffusion marketplace: By embedding physical therapy into primary care, the Veteran can more readily access same day care for musculoskeletal, neurological, and pain complaints. Veterans are also able to receive some prosthetic items in a timelier manner improving function. Earlier access to PT offloads primary care providers, decreases referrals to specialty care and radiology improving efficiency and cost savings.
Selected as national diffusion practice in 2022—from winners of the 7th VHA Shark Tank	
Compassionate contact corps (program to reduce Veteran loneliness through volunteer weekly calls)	Originating facility and DoE fellow: VA Central Ohio health care system (Columbus, Ohio)—Lori Murphy & Anna Giesler
	Number of times adapted by VHA facilities/sites of care: 36 successful, 15 in-progress
	Number of Veterans served as of September 30, 2022: Not yet calculated
	Summary from the VHA diffusion marketplace: Compassionate Contact Corps is a social prescription program that offers friendly phone and video visits between trained volunteers and Veterans. The volunteer calls their matched Veteran once per week to provide compassionate conversation and companionship. This program has been shown to reduce loneliness and improve overall well-being in Veterans.

Practice summaries are quoted (potentially with minor rewording for clarity) from the VHA Diffusion Marketplace as they appeared in March-April of 2023. As of March 31, 2023, the website for the VHA Diffusion Marketplace is Available at: https://marketplace.va.gov/. The number of patients served comes from reports elicited from Diffusion Fellows (person who developed the promising practices) by DoE Diffusion Specialists (DoE staff who lead support of National Diffusion Practices or the Diffusion Marketplace. For this table only, the number of facilities where the National Diffusion Practice has been implemented is the larger number of those recorded in the reports from Diffusion Fellows or Diffusion Specialists mentioned above or in the Diffusion Marketplace. Information to inform the numbers described above was included in an annual report about the numbers of Veterans impacted by and implementation of the National Diffusion Practices that was developed by DoE for the VHA Office of Rural Health.

CPRS, computerized patient record system; HCS, health care system; VA, Veterans affairs; VAMC, Veterans Affairs Medical Center; VHA, veterans health administration promising practices.

DoE's external facilitation is provided to the parent healthcare system/facility or regional team. The local team is led by an Implementing Facility Fellow, the local staff member who guides implementation of the DoE Promising Practice in the “winning” parent healthcare system/facility or region. The Implementing Facility Fellow works with other local team members.

The external facilitation of replication was originally designed to be approximately six months; however, it was extended to 9–12 months due to the COVID-19 pandemic and other contextual factors requiring a longer timeframe or more intensive level of support. Currently, the duration of support remains 9–12 months. The facilitation team includes the Diffusion Fellow who submitted the innovative practice and DoE contractors and staff who provide implementation support. Key ideas from implementation and improvement science are integrated into the facilitation process (e.g., identifying core and adaptable practice components) (31, 54, 55).

The facilitation process begins with the development of implementation plans at a three-day, in-person Base Camp. Base Camp is attended by the Diffusion Fellow (person who developed the DoE Promising Practice) and Implementing Facility Fellow responsible for implementing the innovating practice in the new parent healthcare system/facility. The Base Camp was virtual during the COVID-19 pandemic (following Shark Tanks 6 and 7) and otherwise has been offered in-person. Participants work with a small group facilitator and note taker to develop an implementation plan. The plan defines core and adaptable components of practices, implementation milestones/tasks, key constituents, needed resources, risk mitigation strategies, and initial implementation steps. The teams continue their collaboration for approximately nine to 12 months through: (1) weekly meetings; (2) monitoring the implementation process; (3) solving problems related to implementation barriers or needed practice adaptations; (4) assisting implementing parent healthcare systems/facilities with tasks such as producing promotional materials or toolkits for the practice; (5) providing assistance with tasks such as addressing the impact of VHA regulations on implementation; and (6) identifying national VHA offices and/or constituencies that may engage in broader practice spread throughout the VHA.

### Further diffusion of promising practices

After the nine- to 12-month facilitated replication/implementation phase, DoE leadership and the Office of Healthcare Innovation and Learning determine which of three distinct pathways for further diffusion will be utilized for specific DoE Promising Practices. The decisions are based on a combination of evidence of impact on the problem being addressed, replicability (i.e., portability) in new parent healthcare systems/facilities or locations of care, constituent support and/or cost effectiveness. These specific pathways were introduced for the winners following the 4th Shark Tank.

**Figure 1:**
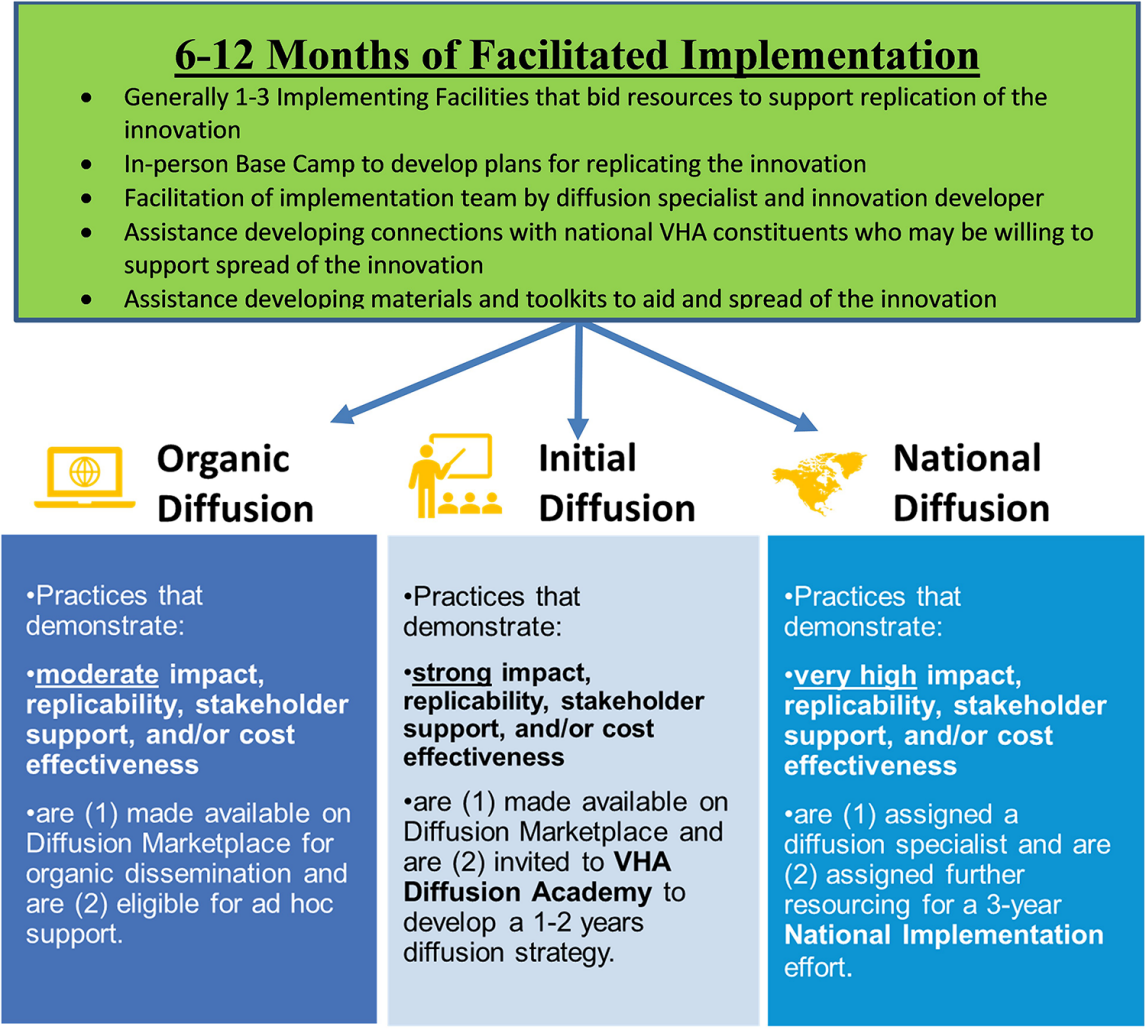
Potential pathway for diffusion following the VHA shark tank.

As shown in Figure 1, a subset of practices is designated for National Diffusion, supported by the DoE staff and contractors. DoE Diffusion Specialists (staff who work for DoE) collaborate with multiple individuals and offices to encourage diffusion of the DoE Promising Practice across the VHA. The individuals with whom a designated Diffusion Specialist works include: the Diffusion Fellows (the Shark Tank winners who developed the DoE Promising Practices), national VHA program offices/constituents, and local parent healthcare systems/facilities to diffuse practices across VHA. Support from DoE is typically provided for three years.

While most practices are VHA Shark Tank Winners, National Diffusion Practices may also be designated as such in collaboration with DoE partners. Thirteen Innovations have been designated National Diffusion Practices. This includes 12 VHA Shank Tank Winners. The 13th practice was transferred to DoE from its sister program that trains frontline staff to develop promising practices, the VHA Innovation Ecosystem (56, 57). These 13 National Diffusion Practices are described in Table 1.

DoE Promising Practices in the second pathway are designated for Initial Diffusion. The developers of these practices are offered the opportunity to receive training through a Diffusion Academy. The Diffusion Academy includes training on the process of refining and spreading practices in cooperation with VHA program offices. As of February 2023, DoE has conducted four Diffusion Academies. The first and fourth Diffusion Academies occurred in person with additional online programing. The second and third Diffusion Academies occurred completely online due to the COVID-19 pandemic. In addition to networking opportunities, participants receive training on topics such as defining practice fidelity, building constituent coalitions, identifying resources and reporting metrics. The goal is to develop a business plan that can be used by the innovation developers to encourage diffusion across the VHA. In addition to VHA Shark Tank winners, Diffusion Academy participants are invited from collaborating partners such as the VHA Innovation Ecosystem (56, 57) and VHA Quality Enhancement Research Initiative (QUERI) (17, 53).

Finally, DoE Promising Practices in the Organic Diffusion Pathway are “packaged” by summarizing information about the practice to encourage teams to organically adopt them if they choose. As with the other two pathways, these promising practices are made available on an online Diffusion Marketplace.

Additional details on DoE and its evolution can also be found in previously published papers (30, 32–42).

### Diffusion marketplace

The Diffusion Marketplace was launched in February of 2020. It became available to the public, outside the VHA, in October of 2021. The Diffusion Marketplace provides an opportunity for healthcare organizations, both inside and outside the VHA, to search for innovations that may address specific challenges. Innovations in the marketplace include practices identified though the DoE Shark Tank and cooperating partners such as the VHA Innovators Network (DoE's sister program within the Innovation Ecosystem, a program that trains frontline staff to develop ideas into promising practices). The Marketplace also hosts innovations developed through VHA's Quality Enhancement Research Initiative (QUERI) (17, 53) and other national VHA program offices. Each entry contains actionable information on implementation resources and processes, along with data on potential innovative practice impacts. The Diffusion Marketplace is also used to track the spread of innovations to new parent healthcare systems/facilities or locations or care across the VHA (i.e., locations that have adopted the innovation). As of April 2023, the Diffusion Marketplace contains entries for 210 innovations and has had 384,435 total users. During January–March 2023, there were 216,406 total page views.

### Evaluation methods

Per regulations outlined in VHA Program Guide 1200.21, the evaluation of the DoE program has been designated a non-research quality improvement activity.

### Organization of the evaluation team

The Spreading Healthcare Access, Activities, Research and Knowledge (SHAARK) partnered evaluation of DoE was funded in April 2017 by the VHA Quality Enhancement Research Initiative (QUERI) (17, 53) following an open, peer-review grant process. The evaluation proposal was co-developed by Diffusion of Excellence leadership and evaluators during a planning period that began in the summer of 2016. The initial 2-year grant has received three subsequent extensions. Since April 1, 2019, the evaluation has been co-funded by the VHA Quality Enhancement Research Initiative (QUERI) and the VHA Office of Rural Health though the DoE. The Office of Rural Health (ORH)/DoE matches Quality Enhncement Research Initiative (QUERI) funds as part of a broader effort in which the Office of Rural Health provides funding to DoE to target the identification and spread of promising innovations that can address the needs of Veterans living in rural areas (e.g., efforts to ease access to care). The Office of Rural Health contributes to SHAARK because of the importance of bringing innovative practices to a third of patients who live in rural areas. The SHAARK evaluation has been primarily guided by the Consolidated Framework for Implementation Research (CFIR) (58) in addition to key concepts from the Weiner Theory of Organizational Readiness for Change (59) and Rogers Theory of Diffusion of Innovation (60).

While not part of the DoE, the SHAARK evaluation is embedded within the DoE team. As part of a learning healthcare system, our embedded evaluation team was highly attentive to the dynamic nature of DoE's activities (39). Our sequential mixed methods design facilitated our use of methods that were responsive to DoE's needs over time (61). We then used RE-AIM to organize our methods and measure/outcome (see Table 2). Each SHAARK extension was planned in collaboration with DoE leaders. SHAARK and DoE have had extensive interactions. This involves meeting at least bi-weekly throughout the evaluation, regularly sharing results, and incorporating DoE feedback into next steps. Evaluators participate in DoE strategic planning meetings to ensure understanding of DoE objectives and discuss evaluation needs and findings.

**Table 2:** Diffusion of excellence evaluation summary organized by the RE-AIM framework.

Evaluation topic	Description of topic	Key outcomes and primary data sources informing listed key outcomes
Program reach	Degree of participation in the diffusion of excellence (DoE)	Key outcomes • In the first eight Shark Tanks (2016–2022) ○ 3,280 Veterans Health Administration (VHA) Shark Tank applications have been submitted ○ 88 DoE promising practices have been designated ○ 140 of 141 of VHA parent health systems/facilities have submitted applications to the VHA Shark Tank ○ 59% of parent healthcare systems/facilities have participated as Sharks Data sources • Tracking of diffusion of excellence program applications and Shark Tank participation
Program effectiveness	Spreading promising practices across the VHA and summary of patients served by individual DoE practices	Key outcomes • The 56 currently tracked DoE Promising Practices (out of the 88 original Shark Tank winners/Doe Promising Practices) have been fully or partially implemented 1,440 times across the VHA (as recorded in the VHA Diffusion Marketplace) • 13 national diffusion practices have been: ○ implemented 966 times at different VHA care locations ○ documented as serving or impacting approximately 978,947 Veterans Data sources • VHA diffusion marketplace (data pulled in March 2023) supplemented by reports from DoE diffusion specialists supporting national diffusion practices obtained in May 2023 • Fiscal year 2022 annual report about Veterans impacted by and implementation of national diffusion practices
Decisions related to DoE program participation and adoption	Considerations relating to adopting practices and participating in diffusion of excellence (as opposed to tracking of the specific number of adoptions, which falls under effectiveness)	Key outcomes • Individuals who develop practices are most commonly responding to a desire to address challenges or opportunities noted as part of their usual work to serve Veterans. • Early results indicated that adoption decisions on the part of parent/health system leaders or directors are most frequently influenced by big picture issues such as how the practice related organizational goals and support of other managers and key constituencies. • Decisions by national VHA leaders to support national spread of practices are also commonly related to big picture issues of how the innovation relates to overall VHA operational goals, support of constituents/other leaders, and the ability to support the effort over the long term (e.g., costs and needed resources). • DoE has taken specific action to develop tools and expectations related to raising awareness of decision makers on what is required to implement innovations and involving staff at a variety of levels in the adoption decision process. ○ While a causal relationship cannot necessarily be drawn, these efforts have been associated with an increase in quality of information provided in Shark Tank bids. Data sources • Semi-structured interviews with diffusion fellows for Shark Tanks 2–3 • Surveys of Sharks conducted following Shark Tanks 2 and 3 • Virtual focus groups conducted with participants in Shark Tanks 3–4 • 2018 semi-structured interviews with VHA • 2021 listening sessions/semi-structured interviews with national VHA leaders • Evaluation of the use of the “QuickView” and “Bid Wishlist” tools to aid facility leaders making adoption decisions during the VHA Shark Tank
Implementation process	Factors influencing implementation success at new facilities	Key outcomes • Following early Shark Tanks, approximately half of facilities replicating practices successfully did so in 6–9 months. • Facilities that have the most successes implementing DoE promising practices align leadership and staff support for the project and needed resources for implementation. • Facilitation helps overcome barriers experienced by facilities. • Some facilities successfully implemented practices with continued work, which has led DoE to expand support for facilitated replication for the original 6 months to 9–12 months. • DoE promising practices that are widely spread across the VHA were able to be successfully implemented during facilitated replication, develop partnerships the VHA national program offices, and focus on processes that can be changed with relatively low need for additional money or resources. Data sources • Semi structured post-implementation telephone interviews Implementing facility fellows for Shark Tanks 1–3, additional implementing parent healthcare system/facility staff for Shark Tanks 2–3, and diffusion fellows for Shark Tanks 2–3. • Longitudinal data collection on the lifecycle of providing practices identified in Shark Tank 4 that occurred from 2018 to 2021, including: artifacts from the DoE's practice selection process, 40 post-facilitated implementation interviews, 2 annual sustainment surveys, 15 lifecycle interviews conducted in 2021, and the evaluation team's periodic reflections about the promising practices.
Maintenance/sustainment of practices	Sustainment of DoE promising practices (including whether practices are still be spread in the VHA)	Key outcomes • 56 of 88 DoE promising practice (64%) are still being actively spread across the VHA, as indicated by inclusion in the VHA diffusion marketplace. • In 2022, 56% of responding facilities that received facilitated replication support following Shark Tanks 1–6 were able to sustain or partially sustain the intervention. • Lack of sustainment can result from practices being integrated with other initiatives/workflows, changes in technology, changes in organizational priorities, and staff having new roles. Data sources • Diffusion marketplace data • Sustainment surveys • Lifecycle interviews conducted in 2021 for promising practices identified during Shark Tank 4, and the evaluation team's periodic reflections about the promising practices.

RE-AIM, reach effectiveness-adoption implementation maintenance.

DoE has undergone reorganization and leadership turnover over the course of the program. The evaluation team has similarly had turnover in membership, but the lead evaluator and many team members have remained consistent. As a result, evaluation priorities have evolved over the years as the program has matured. SHAARK has focused on understanding of factors associated with parent healthcare systems'/facilities' meaningful engagement with DoE, the process of parent healthcare systems/facilities and their leaders choosing to adopt promising practices, factors associated with successful replication of promising practices, and the process of spread of successful promising practices across the VHA.

Except where stated otherwise, most data collection for the evaluation has been organized around either specific Shark Tanks or cohorts of DoE Promising Practices identified through specific Shark Tanks. The focus of the work has evolved over time to match the operational needs of the DoE program and the degree to which key evaluation questions have been addressed. In the sections below on data collection methods, we refer to specific Shark Tanks. These Shark Tanks occurred during the following months: Shak Tank 1 in January 2016, Shark Tank 2 in November 2016, Shark Tank 3 in June 2017, Shark Tank 4 in August 2018, Shark Tank 5 in October 2019, Shark Tank 6 in October 2020, Shark Take 7 in October 2021, and Shark Tank 8 in October 2022.

### Evaluation method 1: tracking Shark Tank applications and practices

Shark Tank applications from Shark Tanks 2–8 have been collected by DoE contractors. Starting with Shark Tank 6, the application process was conducted as part of the Diffusion Marketplace software. Applications for the first Shank Tank, which occurred before the start of the present evaluation, are not available. Applications include information on how the innovation addresses VHA priorities, specific groups of individuals that are targeted, descriptions of the innovation, required resources, and evidence that the innovation has previously had a positive impact on a specific problem. For Shark Tanks 2–7, we report the priorities represented by Shark Tank applications and winners, including priorities of specific interest to rural treatment locations.

Adoptions/replications of specific practices are monitored through the Diffusion Marketplace. The developers of practices included in the Diffusion Marketplace are asked to update specific locations where practices have been implemented. In this paper, we present the total number of adoptions that are listed as fully successful or partially successful/ongoing by the innovators. To be counted in this analysis, an innovative practice must (1) have an entry in the Diffusion Marketplace and (2) have been either a Shark Tank winner or one of the two promising practices that were designated as National Diffusion practices without going through the Shark Tank.

Specific information on the submitting parent healthcare system/facility is available for 2,627 applications linked to a specific parent healthcare system/facility (i.e., applicants not from a VHA VISN, VHA Central Office/Headquarters office, or other office) for Shark Tanks 2–8. Information on parent healthcare system/facility and individual treatment location is available about adoptions/replications in the VHA Diffusion Marketplace. In order to determine the degree to which parent healthcare systems/facilities serving a larger percentage of rural Veterans have submitted applications, we utilized a system developed by the VHA Office of Rural Health that parent healthcare systems/facilities that have more than half of their Veterans residing in rural areas (flags pulled in July 2023). Forty of the 140 parent healthcare systems/facilities located in the United States have more than 50% of patients from rural areas. Rural areas are defined based on the Rural-Urban Commuting Area (RUCA) codes for the location of patients’ homes. RUCA codes are calculated for each census track in the United States for the United States Department of Agriculture (62).

Data from these sources were linked to information about the structure of 141 VHA parent healthcare systems/facilities. This includes 139 VHA parent healthcare system/facility operational complexity designations. VHA gives each facility a complexity score, ranging from 1a (highly complex, multi-specialty parent healthcare system/facility) on through 1b, 1c, 2, to 3 (a small parent healthcare system/facility that mainly provides primary care). Two parent VHA healthcare systems do not have a complexity score. Scores are calculated approximately every 3–4 years based on factors such as breadth of services offered, size, and academic affiliation (63). Another key organizational feature of parent healthcare systems/facilities that was considered is whether the parent healthcare system/facility has access to an innovation specialist (i.e., individual who is employed to help staff develop and pilot innovations as part of the VHA Innovation Ecosystem) (56, 57) to determine if there is an administratively meaningful link between macro-parent healthcare system/facility characteristics and participation in DoE as defined by submitting applications to the VHA Shark Tank (41). The SHAARK evaluation team merged information at the parent healthcare system/facility level because this is the level at which complexity is calculated; there is one healthcare system director and executive leadership team responsible for care and administrative processes across the smaller sites in that system.

Individual treatment locations/facilities were further categorized by rurality. Below, we report percentage of adaptations/replications at urban vs. rural individual treatment locations. This is based on the 1,254 individual treatment locations because a given parent healthcare system/facility can include individual treatment locations in both urban and rural areas. The rural-urban designation is recorded in the official VHA facility list (as of March 14, 2023), based on the Rural-Urban Commuting Area (RUCA) codes for the location of each VHA treatment. RUCA codes are calculated for each census track in the United States for the United States Department of Agriculture (62).

### Evaluation method 2: surveys of key constituents

This evaluation included two surveys of Sharks conducted following Shark Tank cohorts 2 and 3. A survey was sent following the specific Shark Tanks (i.e., a survey was sent to Shark Tank 2 Sharks following Shark Tank 2 conducted in November of 2016 and a survey was sent to Sharks participating in Shark Tank 3 following Shank Tank 3 conducted in June of 2017). Survey questions addressed the Shark experience and asked Sharks who participated in the Shark Tank, regardless of whether their parent healthcare system/facility or region was selected to replicate a DoE promising practice, to rate the importance of specific factors in deciding whether to bid on a practice based on a 5-point scale from completely unimportant to very important (37). Starting with Shark Tank 4, Diffusion Fellows and Implementing Facility Fellows were surveyed before and after a Base Camp (kick-off) meeting, including a question concerning understanding of roles among those involved in operating DoE. Participants in each Diffusion Academy have been surveyed following completion of the program. Finally, Implementing Facility Fellows for the first three Shark Tanks were surveyed in the spring of 2019 asking whether DoE Promising Practices were sustained at their parent healthcare system/facility. A new sustainment survey has been administered on an approximately yearly basis (summer of 2020, 2021, and 2022), adding a new Shark Tank cohort each time (i.e., the 2022 survey included Shark Tanks 1–6).

### Evaluation method 3: qualitative data collected from DoE participants and key constituents

Evaluation team members took notes on process and participant interactions at key DoE events, including Shark Tanks 2–4, DoE Base Camps 2–4, and the DoE Governing Board meeting related to the fourth Shark Tank. These notes were summarized by those taking the notes to allow for real time observation of the DoE process and participant interaction, which allowed the evaluation team to more fully understand the context for evaluation results and provide nearly real-time informal observations to the DoE program. Information was summarized to capture key observations during the events related to DoE process and participant interactions. Virtual focus groups were conducted with Shark Tank participants in Cohorts 3–4, sharing positive aspects of the experience, their ideas for improvement, and how they prepared for the Shark Tank; this information was augmented by responses provided by Shark Tank participants, prompted by questions within an online chat feature (if they participated virtually) or on paper [if they participated in person (during the fourth Shark Tank)]. Written answers to questions were summarized by the evaluation team and presented back to DoE leadership.

Semi structured post-implementation telephone interviews were conducted with Implementing Facility Fellows for Shark Tanks 1–3 (38/45 invited), additional implementing parent healthcare system/facility staff for Shark Tanks 2–3 (40/59), and Diffusion Fellows for Shark Tanks 2–3 [22/23 (27 total people)]. Interviews focused on the process of implementing DoE Promising Practices during the 6–12 month facilitation period as well as DoE processes leading up to implementation, including reasons for practice development (Diffusion Fellows) and participation in DoE. Questions were written to capture constructs from the CFIR, anticipated sustainment, and constructs from the Organizational Readiness for Change (ORC) for interviews with the Diffusion Fellows (58). Analysis of interviews was based on a combination of deductive codes derived from CFIR and ORC constructs and inductive codes for additional categories grounded in the data based on objectives of DoE. Two evaluation team members (CR, AN) performed traditional (CFIR-informed) directed content analysis (64) and resolved coding discrepancies through consensus-based discussions (35). We then aggregated the coded data into a facility-level memo, which we then used to independently rate each CFIR construct for valence (positive or negative influence on implementation) and (2) strength (weak or strong influence on implementation). In a later phase of the evaluation, we adapted the traditional analytic process by developing a rapid CFIR-informed directed content analysis approach. The rapid approach consisted of a primary analyst (interviewer) taking detailed notes immediately after the interview and copying notes into a Microsoft Excel matrix organized by CFIR constructs. A secondary analyst verified and edited the matrix summary while listening to the audio recording for each interview (38). We held weekly consensus-based discussions to resolve analytic discrepancies and refine our analytic approach (35). The matrix was used to facilitate comparisons within and across CFIR constructs and facilities. The matrix also served to integrate relevant qualitative and quantitative data that were collected across different evaluation activities. The traditional and rapid analytic approaches used for interviews have been previously described.

For Shark Tank 4, we sought to evaluate the long-term life cycle of 10 DoE Promising Practices. Longitudinal data collection from 2018 to 2021 included: artifacts from the DoE's Practice selection process, 40 post-facilitated implementation interviews (conducted in 2019), 2 annual sustainment surveys (2020, and 2021), 15 lifecycle interviews conducted in 2021, and the evaluation team's periodic reflections about the Practices. Data were analyzed using content analysis and descriptive statistics to determine: (1). The “problem” of the original facility and the Practice's role as a solution, (2). The implementation outcomes of the original facility (3). The results from participating in the DoE, including implementation and sustainment outcomes from facilitated implementation, and (4). Our primary outcome of interest; the subsequent progression of the practice, i.e., partnership with a VHA National Program Office and current degree to which the practice has been diffused across the VHA. Like the interviews described in the previous paragraph, analysis of qualitative data from interviews and open-ended questions on surveys was based on a combination of deductive codes derived from CFIR constructs and inductive codes for additional categories grounded in the data based on objectives of DoE. The evaluation team performed CFIR-informed directed content analysis (64) and resolved coding discrepancies through consensus-based discussions (35).

While early interviews conducted for SHAARK were coded using transcripts by two-independent coders who met approximately weekly to review discrepancies based on transcripts of the interviews, subsequent interviews were coded in a rapid fashion based on interview notes with confirmation of key points using audio recordings. There was a primary coder who developed a summary memo for the interview and a secondary coder who made edits to the memo. The coders met to discuss discrepancies. The comparison of these processes has been detailed previously (38).

In addition, VHA parent healthcare system/facility directors with at least one year of tenure at their parent healthcare systems/facilities were invited to participate in interviews concerning their concepts of innovation and improvement, decisions to participate in DoE and its sister program the VHA Innovators Network (provides resources and training to staff members working with specific parent healthcare systems/facilities seeking to encourage development of health care innovations at the frontline), medical facility programs to encourage promising practices, and the process of deciding whether to adopt practices. Sixteen leaders were randomly selected, stratified on whether they participated in DoE and/or the Innovators Network. An additional four directors of facilities regarded as highly innovative by DoE were also invited. In the fall of 2018, we interviewed representatives from each invited parent healthcare system/facility, including 20 parent healthcare system/facility interviews that included 28 executive leaders. Detailed notes were taken during interviews. Directed content analysis was performed using *a priori* domains based on the objectives of the interviews described above and additional a posteriori domains identified as salient after data collection. Initial rapid analysis was conducted utilizing templated summaries and a matrix display to facilitate rapid turnaround of results for presentation. This was followed by qualitative content analysis of transcripts from recorded interviews.

Finally, to better understand perceptions of innovations among national VHA leaders who head offices that may partner with innovators towards wider diffusion across the VHA, we partnered with leaders of the VHA Innovation Ecosystem to conduct and analyze a series of listening sessions with 20 VHA national program office leaders conducted in 19 sessions (5 leaders were accompanied by other office staff). These listening sessions were conducted as approximately one-hour, semi-structured interviews conducted in the spring of 2021. They addressed (1) perspectives on innovations; (2) the process of adopting, implementing, and supporting innovation; and (3) opportunities for collaboration with the Innovation Ecosystem, of which DoE is a part. Listening session guides and processes were co-developed by Innovation Ecosystem staff and the SHAARK evaluation team. Innovation Ecosystem staff conducted the listening session and analysis was done by SHAARK evaluation team members. These sessions also focused on opportunities for collaboration among the office and the Innovation Ecosystem, of which DoE is a part. Listening sessions were audio-recorded, transcribed, and then coded by at least 2 evaluators using constructs from the CFIR, along with emerging codes related to leadership perception of innovations.

## Results

Evaluation results are summarized in Table 2 and further described below according to the RE-AIM evaluation framework.

### Reach: DoE program reach

Across the 8 Shark Tanks, there were 3,280 Shark Tank applications submitted (mean = 410; range = 263–622). These applications have led to 88 Shank Tank winners. These winners have been designated as DoE Promising Practices.

The largest number of applications was submitted the two years prior to the COVID-19 pandemic (622 and 591, respectively); the three Shark Tanks occurring since the start of the COVID-19 pandemic have had an average of 338 applications annually. Additional detail below on applications is based on 2,627 applications with corresponding information on submitting information is available on VHA parent healthcare systems/facilities Shark Tanks 2–8. Shark Tank 1 application details are not available. For Shark Tanks 2–8, 140 of 141 VHA parent healthcare systems/facilities (99%) submitted at least one application (mean for all VHA parent healthcare systems/facilities submitting application = 18.7; standard deviation = 14.3; median = 16; range = 1–89).

Larger, more complex VHA parent healthcare systems/facilities have submitted more applications. Specifically, the 39 complexity level 1a parent healthcare systems/facilities in the VHA (highest level of organizational complexity) have submitted 1,137 Shark Tank applications (43.2% of applications; mean for complexity 1a parent healthcare systems/facilities = 29.2; standard deviation = 17.2; median = 26; range = 7–89). This compares to 33 complexity level 3 parent healthcare systems/facilities in the VHA (lowest level of organizational complexity) which have submitted 339 shark tank applications (12.9% of applications; mean for complexity level 3 parent healthcare systems/facilities = 10.3; standard deviation = 8.4; median = 8; range = 1–35). Additionally, 35 VHA Innovators Network (part of the VHA Innovation Ecosystem) member parent healthcare system/facilities (65), which receive support from for developing practices at the frontline of VHA services, submitted Shark Tank applications (mean = 26.6; standard deviation = 19.7; median = 21; range = 7–89) as compared to those sites that are not part of the Innovators Network (mean = 16.0; standard deviation = 11.0; median = 14; range = 1–59).

The 40 parent healthcare system/facilities for whom a majority of Veterans they serve live in rural areas Submitted 547 Shark Tank applications (20.8%), for an average of 13.7 applications per facility (standard deviation = 9.4; median = 12.5; range = 2–43). This compares to an average of 20.8 (standard deviation = 15.4; median = 18.5; range = 1–89) for the other 102 parent healthcare system/facilities.

An additional indication of reach is participation of representatives from parent VHA health care systems/facilities by submitting Shark Tank bids (i.e., fully participating as Sharks). Over half of parent healthcare systems/facilities have participated in at least one Shark Tank by submitting bids for Shark Tanks 1–7 [83/141 (59%)].

### Effectiveness: spreading DoE promising practices across VHA

With a program as complex as DoE, which is focused on many different promising practices, a key indication of success is the ability to identify practices that are then spread across the VHA. As of March 14, 2023, there were a total of 1,440 adaptations/replications of DoE practices across the VHA (i.e., newly adopted at VHA facilities that did not originate the practice). Of the 141 parent healthcare systems/facilities, 136 had at least one adoption/replication (96% of VHA parent healthcare systems/facilities). When considering all 141 parent healthcare systems/facilities, the mean number of adoptions/replications per parent healthcare system/facility was 10.2. The median is 9 (range = 0–33).

Larger, more complex VHA Parent Healthcare Systems/facilities have had a greater number of adoptions/replications of DoE Promising Practices. For example, the 40 complexity level 1a parent healthcare systems/facilities in the VHA have 543 adoptions/replications (38% of adoptions/replications; mean = 13.6; median = 12.5; range = 7–32). This compares to 32 complexity level 3 parent healthcare systems/facilities in the VHA have 229 adoptions/replications (16% of adoptions/replications; mean = 7.2; median = 7; range = 2–15). While there are differences in the number of practices per facility, it should be noted that all of the highest and all of the lowest complexity facilities have adopted at least one DoE Promising Practice.

Looking specifically at rural vs. non-rural locations of care, 180 adoptions recorded in the Diffusion Marketplace happened in rural sites of care (12.5% of adoptions). Twelve percent (56 of 444) rural sites of care had at least one implementation as compared to 30.9% (251 of 810) non-rural sites of care. It is possible that these numbers underestimate actual locations of care if a given parent healthcare system/facility implements a given DoE Promising Practice at more than one location of care and all locations are not listed in the Diffusion Marketplace.

### Effectiveness: number of veterans served by the DoE promising practices

Each practice addresses different problems or challenges facing the VHA. Thus, effectiveness of Shark Tank is conceptualized as the effectiveness of DoE's program broadly, vs. the effectiveness of an individual practice. As a result, it would not be possible to have a combined summary number for all interventions. DoE and Quality Enhancement Research Initiative (QUERI) have funded separate partnered evaluations for a group of the National Diffusion practices. Several other evaluations of individual DoE practices have occurred. The primary purpose of the present evaluation is to examine the impact of the DoE's ability to identify, replicate, and spread promising practices, as opposed to understanding the impact of individual promising practices.

One way the effectiveness of DoE can be conceptualized as the number of Veterans served by the DoE Promising Practices. The National Diffusion Practices track the number of patients they serve based on information in the VHA electronic health record or other systems for indicating who is served. The National Diffusion Practices are estimated to have impacted approximately 978,947 Veterans as of September 30, 2022 and have been implemented at 966 locations of care based on a combination of information recorded in the Diffusion Marketplace in March 2023 and subsequent reports from DoE Diffusion Specialists in May 2023. These practices are summarized in Table 1.

### Adoption: decisions related to DoE program participation and promising practice adoption

Results related to adoption decisions leading to the development of innovations and submission to the Shark Tank competition are based on analysis of qualitative interviews of Diffusion Fellows from Shark Tanks 2–4. We have previously reported that Diffusion Fellows often have an intrinsic motivation to develop and spread promising practices. This is based on a desire to address Veteran or staff needs and frequently results from observation of challenges that are noted on the job. While DoE requires that promising practices be linked to VHA priorities and the impact on performance measures and supporting research are noted, this intrinsic motivation tends to drive the hard work and dedication, frequently in addition to regular job duties, involved in both the development and spread of the DoE promising practices. These individuals collaboratively developed practices with their colleagues and enjoyed strong support from colleagues and leadership.

During Shark Tank pitches and through descriptions of projects, the Shark Tank finalists must convince parent healthcare systems/facility or regional leaders of the potential value of implementing the practice in their parent healthcare system/facility or region. An important aspect of this process is considering what leadership views as a valuable innovation, worth bidding on and committing resources to adopt.

It can be challenging to translate the enthusiasm of a Diffusion Fellow to new sites, due in part to differing perspectives of senior VHA parent healthcare system/facility leaders and of frontline staff. Interviews with 28 facility leaders from 20 facilities (primarily parent healthcare system/facility directors) conducted in 2019 indicate that health system leaders look for innovations with practical connections to the overall objectives and needs of the system. This is in line with findings we have previously reported that senior leaders have broad objectives in mind when deciding to participate in programs such as DoE and when making specific practice adoption decisions (37). Based on qualitative interviews of parent healthcare system/facility executive leaders, the perception of innovative culture, the opportunity to share and implement promising practices, and potential for improving parent healthcare system/facility performance metrics were the top reasons cited by leadership for deciding to participate in DoE. Further, leaders primarily base decisions to implement particular practices on whether the practice aligns with the organization's strategic plan and overall VHA priorities, contributes to stronger engagement and constituent buy-in, and affects quality and safety.

These findings from parent healthcare system/facility leaders are in line with results of qualitative listening sessions with leaders of national VHA program offices. These individuals tend to define innovation as the implementation of new evidence-based interventions that improve the ability for the organization to respond to the needs of Veterans. When making decisions about whether to support national spread of innovations, they consider topics such as availability of resources to spread and sustain innovation, networks to support broad implementation, fit with organizational culture, and how the innovation fits into the priorities and needs of key players and change agents.

The SHAARK evaluation team worked with DoE leadership to develop a “QuickView” tool, displaying major considerations for implementation (e.g., required staffing, anticipated time to implement, need for information technology support) in a grid format to facilitate comparison across practices. This tool was implemented for the 4th Shank Tank. Subsequently, the SHAARK team added a Bid “Wishlist” in which innovation developers described minimum required resources necessary to implement the innovation and also described additional resources that, while not required, would make implementation easier if available. As detailed elsewhere (42), an analysis of Shank Tank applications from the 2nd to 6th Shark Tanks found that introduction of the QuickView and Wishlist tools was followed by an increase in the degree to which Shark Tank bids directly addressed the need for the innovative practice at their parent healthcare system/facility. Bids providing details on specific resources committed also increased in the years after these tools were introduced.

Healthcare system and facility leaders are responsible for considering the big picture at their local institutions, but this high-level perspective does not always address the concerns of the frontline staff who must accomplish key goals. As we have previously reported, qualitative findings from those originating and from those replicating innovations indicate that if leaders were not familiar with key details regarding the innovations being implemented, implementation could be much more difficult. Lack of familiarity on the part of leadership could also translate to decreased engagement by frontline staff.

### Implementation: implementation of DoE promising practices

Early in the evaluation process, we noted the importance of involving key constituents at all levels, throughout implementation. Initially Implementing Facility Fellows were relatively uninvolved in the Shark Tank Competition process. For example, systematic observations of team interactions during the 2nd, 3rd, and 4th in-person DoE Base Camps revealed situations in which Implementing Facility Fellows responsible for leading the implementation in their parent healthcare systems/facilities or regions did not have a full awareness of available resources promised by their directors or of the underlying leadership goals. This lack of knowledge led some DoE participants to be apprehensive about beginning involvement with the DoE process. Lack of participation by key constituents in the VHA parent healthcare system/facility or region can decrease the degree to which these constituents feel they are actively bringing the needed DoE Promising Practice to their colleagues as opposed to having it thrust upon them. This is a key reason for the development of the QuickView and Bid Wishlist tools mentioned above. These tools were intended to encourage involvement of staff in the bidding process and to focus Sharks on considerations of resources needed to make replication of DoE Promising Practices successful.

Further, the need for role clarity is an area of continuous quality improvement for DoE. Surveys completed prior to the Base Camp indicate that some participants enter the process not fully aware of what will happen during the replication and what may result for them and their DoE Promising Practices after completion of the nine-12 month implementation process. Surveys after the Base Camp indicate that most participants report that the Base Camp is a key place for not only developing plans to implement/replicate the DoE Promising Practice, but also to clarify their own roles going forward. In addition to the focus on the Base Camp, DoE has responded to the need for role clarification by engaging in earlier interaction with DoE participants and requiring a writing participation agreement between leaders of participating parent healthcare systems/facilities and DoE.

As we have previously reported (35), barriers to implementation can generally be overcome during the intensive six- to 12-month facilitated implementation, unless an insurmountable barrier is encountered (e.g., barriers such as lack of necessary infrastructure, resources, or staff). Based on interviews with implementing facilities from Shark Tanks 2–3, approximately half of implementing parent healthcare systems/facilities successfully implemented during the approximately 6 month facilitated replication/implementation process (35), with the remainder being partially successful or unable to reach implementation goals. However, a number of parent healthcare systems/facilities were able to complete implementation with additional time. This finding, along with subsequent circumstances related to the COVID-19 pandemic, encouraged DoE to extend the period of active facilitated implementation to nine-12 months.

In depth analysis of the lifecycle of 10 DoE Promising Practices identified through the 4th Shark Tank provides important information about the link between the initial replication of the practices through the DoE process and the degree of eventual spread/diffusion across the VHA. Practices with extensive diffusion (defined as diffusion to 10 or more parent healthcare systems/facilities or two or more VHA regions) successfully completed facilitated implementation and were sustained at each timepoint thereafter, whereas 2 of the 3 Practices with minimal diffusion (less than 10 parent healthcare systems/facilities or less than 1 VHA region) did not successfully implement nor sustain at their facilitated implementation facilities. Additionally, DoE Promising Practices that had a VHA national program office as a funding partner were more likely to diffuse across VHA. National program offices, after connecting to DoE Promising Practices through DoE, function as a major source of support for diffusion, acting as either a practice endorser (no funding), small-scale funding partner (funding select parent healthcare systems/facilities), or broad-scale funding partner (funding for a dedicated team to aid with diffusion). Thirty percent (3/10) of the DoE Promising Practices from the 4th Shark Tank had a national program office acting as a broad-scale funding partner, which supported extensive diffusion. We found the likelihood of an innovation receiving such a partner depends primarily on the national program office's interests, priorities, and resources at that time. For example, though clinical interventions often have an excellent evidence base, 2 of the 3 DoE Promising Practices with extensive diffusion were process improvements. Clinical interventions were found less likely to be selected for national program office partnership due to their cost. These findings are in line with findings from qualitative listening sessions with national program office leaders described in the adoption section above.

### Maintenance: sustainment of DoE promising practices

Sustainment of practices is defined two ways. First, practices are considered active (i.e., sustained) if they are currently being tracked in the Diffusion Marketplace. Of 88 Shark Tank winners (i.e., DoE Promising Practices), 56 (64%) practices are being actively followed in the Diffusion Marketplace. There are three primary reasons for practices no longer being actively followed. First, some practices are merged with other initiatives. For example, a practice that focused on chaplain-led groups to address the challenge of moral injury among Veterans has been merged with an initiative to spread the use of related groups that are co-led by healthcare chaplains and mental health providers. This initiative includes the developer of the DoE promising practice and is a partnership among VHA program offices (e.g., the VHA Integrative Mental Health Program), Quality Enhancement Research Initiative (QUERI), and DoE (55, 67). Second, new technology or VHA initiatives have incorporated key aspects of previous innovations. For example, an early DoE Promising Practice was an icon on VHA desktops that led to a form to report annual flu shots. As new technology and vaccine reporting initiatives have occurred, this reporting function is now addressed through other technology. Finally, as with any organization, staff turnover occurs resulting in the developer of a practice leaving the VHA before it has fully gained traction.

A second key indication of the sustainment of the practice is whether it was sustained at the original Implementing Facility. While these are select sites that had at least initially indicated they had resources to implement the practice, the ability to sustain the practice after facilitated replication is an indication of the potential feasibility of sustainment at other parent healthcare systems/facilities or locations of care. This is important because it is unfortunately not feasible to ascertain sustainment of practices across all instances in which a practice has been adopted. While the percentage of responding facilities indicating practices have been sustained has gone from 72% based on the 2020 implementation survey to 56% on the 2022 survey. This may be somewhat expected because time since facilitated replication has increased, meaning that there is more time for circumstances to occur that can impact the sustainment of the innovative practice (e.g., organizational-level impacts of the COVID-19 pandemic) (40, 68).

## Discussion

This paper presents an overall summary of a long-term, nearly seven-year, embedded evaluation of VHA's DoE Shark Tank program. The findings demonstrate how evaluation teams can utilize the RE-AIM framework to help understand the broad impact of an innovation program beyond summarizing the impact of individual projects. While evaluation of these individual projects/innovations is certainly important, so is the holistic evaluation of programs such as DoE to establish an infrastructure and process to identify, replicate, and spread innovations across health systems. This holistic evaluation approach is key to realizing the potential goals of the learning health system model, especially as healthcare systems become larger and larger entities.

With 140 of 141 parent healthcare systems/facilities submitting Shark Tank applications and 136 of 141 parent healthcare system/facilities reporting adoption of DoE practices, the program has a very broad reach across the VHA. The program has identified more than 3,000 potential innovations that have applied to the VHA Shark Tank competition. As recorded in the VHA Diffusion Marketplace, DoE Promising Practices have been successfully or partially adopted 1,440 times across the VHA.

Both during initial replication of promising practices in new locations and when decisions are being made about the degree to which spread of the innovation will be supported nationally across VHA, we noted the importance of aligning big-picture considerations related to organizational goals, constituent support, and costs of implementation that motivate executive leaders with the needs of frontline staff who must address the reality of implementing new innovations on the ground.

DoE's efforts are closely tied to findings from the SHAARK evaluation team, exemplifying a learning healthcare system. Over the course of the eight Shark Tanks conducted by DoE, the program has evolved, based in part on findings from the evaluation. DoE has adapted by enhancing participation strategies for those engaged in the Shark Tank and DoE process and by connecting with staff at multiple levels within healthcare facilities. Examples include DoE efforts to educate participants on expectations earlier in the process, use of participation agreements, use of tools such as the QuickView and Bid Wishlist, and extension of the facilitated support period. Along with these changes, ongoing efforts to improve role clarification for the various participants in DoE remains a priority. These enhancements have been associated with an improvement in the quality of Shark Tank bids.

Unlike evaluating a single intervention or innovation where there is a specific outcome to be evaluated (e.g., a clinical parameter, process, or cost), the goal of the present evaluation has been to determine if DoE is an effective mechanism for identifying innovations that are occurring across more than 1,200 locations of care, helping to determine if impactful replication in new sites is feasible, and spreading highly impactful processes across the VHA. Overall, the DoE has been able to accomplish these goals. Separate evaluations have found that a number of these practices are in fact effective at achieving the specific practice goals.

The VHA experiences reported here also demonstrate the role of an embedded, long-term evaluation of large and evolving programs. When the evaluation started, the focus was on understanding the Shark Tank process. As noted above, the DoE evolved by making numerous changes to its processes based on evaluation findings. Going forward, the SHAARK evaluation will be focused on helping develop tools to enhance innovation infrastructure at facilities across the VHA, to increase both participation in the Shark Tank process and adoption of DoE Promising Practices at sites with less participation (including a focus on treatment locations in rural areas). Additionally, this evaluation will enhance the tracking of innovation adoptions across the VHA and seek to understand how to support the sustainment of these innovations once adopted.

Along with other parts of the VHA Office of Healthcare Innovations and Learning, DoE is predicated on the fact that learning health systems need to both support the grassroots, bottom up, ideas of clinical and administrative staff across the health system and provide centralized, top-down support structure for those seeking to bring healthcare innovations to Veterans. This combination, along with the continuing evaluation of DoE, demonstrate VHA's commitment to being a learning health system with leadership support for continuing to learn how to better serve Veterans, empowering staff to solve problems in new ways, and evaluating the impact of programs designed to support the learning health system. These broad efforts are particularly important for rural healthcare facilities. VHA is the US's largest integrated national healthcare system, providing care to 6.75 million patients, a third of whom reside in rural areas.

Collectively, DoE's efforts advance the care of rural patients. However, future evaluation efforts are needed to more fully explicate potential difference in how organizational factors influence the organization of programs to support the development and implementation of innovations within healthcare facilities across rural, suburban, and urban areas. While the present evaluation provides information on the degree to which DoE has been able to support adoption of new innovations in rural areas, future evaluation work is needed to furth explicate differences in the way DoE and other related programs impact the organizational support of innovations in rural and non-rural facilities.

### Limitations

This work represents a non-research, quality improvement evaluation of a program within the VHA. However, the evaluation is based on the methods of implementation and improvement science. While the VHA differs from private-sector health systems and those in other parts of the world, many of the opportunities and challenges observed though the evaluation of the DoE are observed in other large health systems seeking to understand the impact and structure of many new non-research innovation programs that have been established over the last decade or so (69). A detailed assessment of the effectiveness (e.g., clinical, administrative, or economic outcomes) and implementation outcomes (e.g., practice fidelity) of specific DoE Promising Practice on outcomes is beyond the scope of this paper and the SHAARK evaluation. This paper also represents an updated summary of a long-running evaluation of DoE. As a result, we note that certain findings represent results from in-depth analyses of specific practices or Shark Tank cohorts. At times, this is due to the fact that DoE utilizes information and then evaluators and the DoE determine new aspects of the program that must be evaluated. As a result, all data elements are not collected across all time points. The result being that longitudinal comparisons across all elements of the RE-AIM framework are not possible. Additionally, specific information collected by DoE has evolved with the program and must account for feasibility of capturing information for operational and evaluation purposes as opposed to research purposes. For example, the Diffusion Marketplace was launched in February 2020, just prior to the beginning of the height of societal disruptions caused by the COVID-19 pandemic. While the Diffusion Marketplace capabilities have expanded over time, it would not be feasible to capture all information that we would like to have for evaluation purposes (e.g., the exact date a new facility adopted a DoE Promising Practice). Finally, other analyses were done periodically, and we present information from the most recently completed analysis because we are presenting a summary of findings for the overall program.

As noted above, data were collected and analyzed based in large part on the changing operational needs of the DoE program. This is because the evaluation is both long-term in nature (collaboration beginning in 2016) and embedded within the program (i.e., while evaluators are independent, they work closely with the DoE program) (39). We consider this a strength of the evaluation in that it means that methodologically rigorous evaluation findings are highly recent and utilized by DoE. The evaluation is also able to respond to changes in the program and healthcare system (e.g., development of the Diffusion Marketplace and the realities of the COVID-19 pandemic). However, this presents a limitation in that we were not then able to use the exact same methods and time-points for collecting data over the entire course of the still ongoing evaluation work, limiting longitudinal analyses.

### Positionality of the evaluators

We have previously published on key issues that must be balanced in conducting embedded evaluations, including the degree to which evaluators are involved in operational discussions, present information to program leaders and participants, and the degree to which evaluators are involved in different aspects of the program and its sponsoring office (39). SHAARK represents a highly embedded evaluation. While we believe that this has been important in both interpreting evaluation findings and producing information useful to the program, we recognize that we must specifically consider ways in which evaluators maintain objectivity and reduce potential bias. Authors on this paper are both evaluators and DoE staff or contractors who have collaborated. Additionally, DoE, along with the VHA Office or Rural Health and VHA Quality Enhancement Research Initiative (QUERI) provide the funding for this evaluation. We have also partnered with DoE and its parent organizations, the VHA Innovation Ecosystem and Office of Healthcare Innovation and Learning, on numerous initiatives. Evaluators have never been told not to publish or present findings outside the VHA. Especially when presenting a summary of findings about the program as a whole over the years, we believe it is important to be clear about the ways in which evaluators and program personnel collaborate.

## Conclusion

DoE's infrastructure and processes have successfully identified thousands of promising practices being used on the frontlines of VHA care that are sustainable and have been spread across the nationwide system. It is important to continually consider how to expand these efforts in rural locations of care, which may not have access to the same infrastructure for supporting innovations as urban locations of care. The program and its evaluation process offer other large learning health systems an example of a program that has matured and evolved over the course of more than seven years, including maintaining momentum during the COVID-19 pandemic.

## Data Availability

The datasets presented in this article are not readily available because Data were generated as part of a non-research quality improvement evaluation conducted within the United States Department of Veterans Affairs. You may contact the corresponding author to discuss potential availability of specific data elements. Availability of data is governed by applicable regulations concertning availability of data from the United States Department of Veterans Affairs. Requests to access the datasets should be directed to George L. Jackson, Ph.D., MHA, george.jackson3@va.gov; george.jackson@utsouthwestern.edu.

## References

[1] Berwick DM Nolan TW Whittington J. The triple aim: care, health, and cost. Health Aff (Millwood). (2008) 27(3):759–69. 10.1377/hlthaff.27.3.759 18474969

[2] Jackson GL Powers BJ Chatterjee R Bettger JP Kemper AR Hasselblad V The patient centered medical home. A systematic review. Ann Intern Med. (2013) 158(3):169–78. 10.7326/0003-4819-158-3-201302050-00579 24779044

[3] Corrigan JM Clancy CM. Assessing progress in health care quality through the Lens of COVID-19. JAMA. (2020) 324(24):2483–4. 10.1001/jama.2020.17392 33351050

[4] Bodenheimer T Sinsky C. From triple to quadruple aim: care of the patient requires care of the provider. Ann Fam Med. (2014) 12(6):573–6. 10.1370/afm.1713 25384822 PMC4226781

[5] Sikka R Morath JM Leape L. The quadruple aim: care, health, cost and meaning in work. BMJ Qual Saf. (2015) 24(10):608–10. 10.1136/bmjqs-2015-004160 26038586

[6] Nieuwsma JA O'Brien EC Xu H Smigelsky MA, VISN 6 MIRECC Workgroup, HERO Research Program, et al. Patterns of potential moral injury in post-9/11 combat veterans and COVID-19 healthcare workers. J Gen Intern Med. (2022) 37(8):2033–40. 10.1007/s11606-022-07487-4 35381899 PMC8982664

[7] Everett CM Docherty SL Matheson E Morgan PA Price A Christy J Teaming up in primary care: membership boundaries, interdependence, and coordination. JAAPA. (2022) 35(2):1–10. 10.1097/01.JAA.0000805840.00477.58 34985006 PMC9869344

[8] Greilich PE Kilcullen M Paquette S Lazzara EH Scielzo S Hernandez J Team FIRST framework: identifying core teamwork competencies critical to interprofessional healthcare curricula. J Clin Transl Sci. (2023) 7(1):e106. 10.1017/cts.2023.27 37250989 PMC10225264

[9] Starfield B. Basic concepts in population health and health care. J Epidemiol Community Health. (2001) 55(7):452–4. 10.1136/jech.55.7.452 11413173 PMC1731926

[10] Washington AE Coye MJ Boulware LE. Academic health systems’ third curve: population health improvement. JAMA. (2016) 315(5):459–60. 10.1001/jama.2015.18550 26836726

[11] Leykum LK Penney LS Dang S Trivedi RB Noël PH Pugh JA Recommendations to improve health outcomes through recognizing and supporting caregivers. J Gen Intern Med. (2022) 37(5):1265–9. 10.1007/s11606-021-07247-w 34981348 PMC8722428

[12] Van Houtven CH. Standing up for my sister. Health Aff (Millwood). (2022) 41(10):1523–7. 10.1377/hlthaff.2022.00780 36190889

[13] Vohra SS Rajupet SR Kaminski MA White MA Fagerlin A Ellerbeck EF. Evolution of population health within US schools of medicine and academic medical centers. Popul Health Manag. (2023) 26(4):268–74. 10.1089/pop.2023.0047 37590082

[14] Atkins D Kilbourne AM Shulkin D. Moving from discovery to system-wide change: the role of research in a learning health care system: experience from three decades of health systems research in the Veterans health administration. Annu Rev Public Health. (2017) 38:467–87. 10.1146/annurev-publhealth-031816-044255 28125386

[15] Committee on the Learning Health Care System in America; Institute of Medicine. Best care at lower cost: The path to continuously learning health care in America. Smith M Saunders R Stuckhardt L McGinnis JM, editors. Washington (DC): National Academies Press (US) (2013). 10.17226/13444 24901184

[16] Palakshappa D Miller DP Jr. Rosenthal GE. Advancing the learning health system by incorporating social determinants. Am J Manag Care. (2020) 26(1):e4–6. 10.37765/ajmc.2020.42146 31951360

[17] Kilbourne AM Goodrich DE Miake-Lye I Braganza MZ Bowersox NW. Quality enhancement research initiative implementation roadmap: toward sustainability of evidence-based practices in a learning health system. Med Care. (2019) 57(Suppl 10 Suppl 3):S286–93. 10.1097/MLR.0000000000001144 31517801 PMC6750196

[18] Kilbourne AM Braganza MZ Bowersox NW Goodrich DE Miake-Lye I Floyd N Research lifecycle to increase the substantial real-world impact of research: accelerating innovations to application. Med Care. (2019) 57(Suppl 10 Suppl 3):S206–12. 10.1097/MLR.0000000000001146 31517789 PMC6750195

[19] Masica AL Velasco F Nelson TL Medford RJ Hughes AE Pandey A The Texas health resources clinical scholars program: learning healthcare system workforce development through embedded translational research. Learn Health Syst. (2022) 6(4):e10332. 10.1002/lrh2.10332 36263262 PMC9576247

[20] Kilbourne AM Schmidt J Edmunds M Vega R Bowersox N Atkins D. How the VA is training the next-generation workforce for learning health systems. Learn Health Syst. (2022) 6(4):e10333. 10.1002/lrh2.10333 36263263 PMC9576233

[21] Lozano PM Lane-Fall M Franklin PD Rothman RL Gonzales R Ong MK Training the next generation of learning health system scientists. Learn Health Syst. (2022) 6(4):e10342. 10.1002/lrh2.10342 36263260 PMC9576226

[22] Dzau VJ Yoediono Z Ellaissi WF Cho AH. Fostering innovation in medicine and health care: what must academic health centers do? Acad Med. (2013) 88(10):1424–9. 10.1097/ACM.0b013e3182a32fc2 23969357

[23] Ellner AL Stout S Sullivan EE Griffiths EP Mountjoy A Phillips RS. Health systems innovation at academic health centers: leading in a new era of health care delivery. Acad Med. (2015) 90(7):872–80. 10.1097/ACM.0000000000000679 25738387

[24] Asch DA Terwiesch C Mahoney KB Rosin R. Insourcing health care innovation. N Engl J Med. (2014) 370(19):1775–7. 10.1056/NEJMp1401135 24806157

[25] Bates DW Sheikh A Asch DA. Innovative environments in health care: where and how new approaches to care are succeeding. Health Aff (Millwood). (2017) 36(3):400–7. 10.1377/hlthaff.2016.1311 28264940

[26] Li J Williams MV Page C Cassis L Kern PA DiPaola RS. The value of innovation to implementation program (VI^2^P): a strategic approach to aligning and leveraging academic research and clinical care missions. Learn Health Syst. (2019) 3(4):e10199. 10.1002/lrh2.10199 31641687 PMC6802527

[27] Rotenstein LS Wickner P Hauser L Littlefield M Abbett S Desrosiers J An academic medical center-based incubator to promote clinical innovation and financial value. Jt Comm J Qual Patient Saf. (2019) 45(4):259–67. 10.1016/j.jcjq.2018.12.004 30665836

[28] Psek WA Stametz RA Bailey-Davis LD Davis D Darer J Faucett WA Operationalizing the learning health care system in an integrated delivery system. EGEMS (Wash DC). (2015) 3(1):1122. 10.13063/2327-9214.1122 25992388 PMC4434917

[29] Frakt AB Prentice JC Pizer SD Elwy AR Garrido MM Kilbourne AM Overcoming challenges to evidence-based policy development in a large, integrated delivery system. Health Serv Res. (2018) 53(6):4789–807. 10.1111/1475-6773.12986 29862494 PMC6232400

[30] Elnahal SM Clancy CM Shulkin DJ. A framework for disseminating clinical best practices in the VA health system. JAMA. (2017) 317(3):255–6. 10.1001/jama.2016.18764 28114562

[31] Jackson GL Cutrona SL Kilbourne A White BS Everett C Damschroder LJ. Implementation science: helping healthcare systems improve. JAAPA. (2020) 33(1):51–3. 10.1097/01.JAA.0000615508.92677.66 31880652

[32] Vega R Jackson GL Henderson B Clancy C McPhail J Cutrona SL Diffusion of excellence: accelerating the spread of clinical innovation and best practices across the nation’s largest health system. Perm J. (2019) 23:309. 10.7812/TPP/18.309 PMC6836565 31634112

[33] Clancy C. Creating world-class care and service for our nation’s finest: how Veterans health administration diffusion of excellence initiative is innovating and transforming veterans affairs health care. Perm J. (2019) 23:301. 10.7812/TPP/18.301 PMC6836558 31634111

[34] VA health care, efforts to prioritize and translate research into clinical practice (United States Government Accountability Office, GAO-20-211). (2020).

[35] Nevedal AL Reardon CM Jackson GL Cutrona SL White B Gifford AL Implementation and sustainment of diverse practices in a large integrated health system: a mixed methods study. Implement Sci Commun. (2020) 1(1):61. 10.1186/s43058-020-00053-1 32885216 PMC7427879

[36] Vega RJ Kizer KW. VHA’s innovation ecosystem: operationalizing innovation in health care. NEJM Catalyst. (2020) 1 (6). 10.1056/CAT.20.0263

[37] Jackson GL Cutrona SL White BS Eardon CM Orvek E Nevedal AL Merging implementation practice and science to scale up promising practices: the Veterans health administration (VHA) diffusion of excellence (DoE) program. Jt Comm J Qual Patient Saf. (2021) 47(4):217–27. 10.1016/j.jcjq.2020.11.014 33549485

[38] Nevedal AL Reardon CM Opra Widerquist MA Jackson GL Cutrona SL White BS Rapid versus traditional qualitative analysis using the consolidated framework for implementation research (CFIR). Implement Sci. (2021) 16(1):67. 10.1186/s13012-021-01111-5 34215286 PMC8252308

[39] Jackson GL Damschroder LJ White BS Henderson B Vega RJ Kilbourne AM Balancing reality in embedded research and evaluation: low vs high embeddedness. Learn Health Syst. (2022) 6(2):e10294. 10.1002/lrh2.10294 35434356 PMC9006533

[40] Reardon CM Damschroder L Opra Widerquist MA Arasim M Jackson GL White B Sustainment of diverse evidence-informed practices disseminated in the Veterans health administration (VHA): initial development and piloting of a pragmatic survey tool. Implement Sci Commun. (2023) 4(1):6. 10.1186/s43058-022-00386-z 36647162 PMC9842210

[41] Kaitz J DeLaughter K Deeney C Cutrona SL Hogan TP Gifford AL Leveraging organizational conditions for innovation: a typology of facility engagement in the Veterans health administration shark tank-style competition. Perm J. (2023) 27:1–8. 10.7812/TPP/22.154 PMC10266856 36946078

[42] Cutrona SL White L Miano D Damschroder LJ Hogan TP Gifford AL Supporting veteran’s administration medical center directors’ decisions when adopting innovative practices: development and implementation of the “QuickView” and “WishList” tools. Perm J. (2023) 27(3):79–91. 10.7812/TPP/23.008 37545198 PMC10502382

[43] Glasgow RE Vogt TM Boles SM. Evaluating the public health impact of health promotion interventions: the RE-AIM framework. Am J Public Health. (1999) 89(9):1322–7. 10.2105/AJPH.89.9.1322 10474547 PMC1508772

[44] Glasgow RE McKay HG Piette JD Reynolds KD. The RE-AIM framework for evaluating interventions: what can it tell US about approaches to chronic illness management? Patient Educ Couns. (2001) 44(2):119–27. 10.1016/S0738-3991(00)00186-5 11479052

[45] Glasgow RE Harden SM Gaglio B Rabin B Smith ML Porter GC RE-AIM planning and evaluation framework: adapting to new science and practice with a 20-year review. Front Public Health. (2019) 7:64. 10.3389/fpubh.2019.00064 30984733 PMC6450067

[46] Peracca SB Jackson GL Lamkin RP Mohr DC Zhao M Lachica O Implementing teledermatology for rural veterans: an evaluation using the RE-AIM framework. Telemed J E Health. (2021) 27(2):218–26. 10.1089/tmj.2020.0013 32343924

[47] Vaughan CP Brown RT Hastings SN Makris UE Forman DE. Veterans health administration research in aging: opportunities for high impact across the academic career. J Am Geriatr Soc. (2023) 71:3001–4. 10.1111/jgs.18393 37093614 PMC10693935

[48] Meffert BN Morabito DM Sawicki DA Hausman C Southwick SM Pietrzak RH US Veterans who do and do not utilize veterans affairs health care services: demographic, military, medical, and psychosocial characteristics. Prim Care Companion CNS Disord. (2019) 21(1):18m02350. 10.4088/PCC.18m02350 30677266 PMC6352911

[49] Kangovi S Mitra N Grande D White ML McCollum S Sellman J Patient-centered community health worker intervention to improve posthospital outcomes: a randomized clinical trial. JAMA Intern Med. (2014) 174(4):535–43. 10.1001/jamainternmed.2013.14327 24515422

[50] Crowley MJ Edelman D McAndrew AT Kistler S Danus S Webb JA Practical telemedicine for veterans with persistently poor diabetes control: a randomized pilot trial. Telemed J E Health. (2016) 22(5):376–84. 10.1089/tmj.2015.0145 26540163

[51] Hastings SN Sloane R Morey MC Pavon JM Hoenig H. Assisted early mobility for hospitalized older veterans: preliminary data from the STRIDE program. J Am Geriatr Soc. (2014) 62(11):2180–4. 10.1111/jgs.13095 25354909 PMC4264567

[52] Woodbury MG Kuhnke JL. Evidence-based practice vs. evidence-informed practice: what’s the difference? Wound Care Can. (2014) 12(1):26–9.

[53] Braganza MZ Kilbourne AM. The quality enhancement research initiative (QUERI) impact framework: measuring the real-world impact of implementation science. J Gen Intern Med. (2021) 36(2):396–403. 10.1007/s11606-020-06143-z 32875498 PMC7878630

[54] Aarons GA Green AE Palinkas LA Self-Brown S Whitaker DJ Lutzker JR Dynamic adaptation process to implement an evidence-based child maltreatment intervention. Implement Sci. (2012) 7:32. 10.1186/1748-5908-7-32 22512914 PMC3436717

[55] Smigelsky MA Nieuwsma JA Meador K Vega RJ Henderson B Jackson GL. Dynamic diffusion network: advancing moral injury care and suicide prevention using an innovative model. Healthc (Amst). (2020) 8(3):100440. 10.1016/j.hjdsi.2020.100440 32919579 PMC7405892

[56] Vashi AA Orvek EA Tuepker A Jackson GL Amrhein A Cole B The Veterans health administration (VHA) innovators network: evaluation design, methods and lessons learned through an embedded research approach. Healthc (Amst). (2021) 8(Suppl 1):100477. 10.1016/j.hjdsi.2020.100477 34175094 PMC8244154

[57] Amrhein A. Investing in the front line: leading a cultural innovation revolution. J Healthc Manag. (2021) 66(5):332–5. 10.1097/JHM-D-21-00196 34494998

[58] Damschroder LJ Aron DC Keith RE Kirsh SR Alexander JA Lowery JC. Fostering implementation of health services research findings into practice: a consolidated framework for advancing implementation science. Implement Sci. (2009) 4:50. 10.1186/1748-5908-4-50 19664226 PMC2736161

[59] Weiner BJ. A theory of organizational readiness for change. Implement Sci. (2009) 4:67. 10.1186/1748-5908-4-671748-5908-4-67 19840381 PMC2770024

[60] Rogers EM. Diffusion of innovations. 4th ed. New York, NY: The Free Press (1995).

[61] Fetters MD Curry LA Creswell JW. Achieving integration in mixed methods designs-principles and practices. Health Serv Res. (2013) 48(6 Pt 2):2134–56. 10.1111/1475-6773.12117 24279835 PMC4097839

[62] Rural-Urban Commuting Area Codes. *United States Department of Agriculture*. Available at: https://www.ers.usda.gov/data-products/rural-urban-commuting-area-codes/ (Accessed May 3, 2023).

[63] National Academies of Sciences E, and Medicine. Facilities staffing requirements for the veterans health administration resource planning and methodology for the future (2020). Washington, DC: The National Academies Press (2020). 136. 32293829

[64] Hsieh HF Shannon SE. Three approaches to qualitative content analysis. Qual Health Res. (2005) 15(9):1277–88. 10.1177/1049732305276687 16204405

[65] Vashi A Jackson GL Tuepker A Cutrona S. Evaluation of innovators network aims to understand why innovations succeed, and if they can be scaled and spread. Forum. (2018) 21(2):10–2.

[66] Smigelsky MA Trimm V Meador K Jackson GL Wortmann JH Nieuwsma JA. Core components of moral injury groups co-facilitated by mental health providers and chaplains. Spiritual Clin Pract (Wash D C). (2022) 9(3):159–74. 10.1037/scp0000297 37360983 PMC10288643

[67] Chambers DA Glasgow RE Stange KC. The dynamic sustainability framework: addressing the paradox of sustainment amid ongoing change. Implement Sci. (2013) 8:117. 10.1186/1748-5908-8-117 24088228 PMC3852739

[68] Lineen J. Hospital consolidation: “safety in numbers” strategy prevails in preparation for a value-based marketplace. J Healthc Manag. (2014) 59(5):315–7. 25647949

